# A retrospective analysis of ezrin protein and mRNA expression in breast cancer: Ezrin expression is associated with patient survival and survival of patients with receptor‐positive disease

**DOI:** 10.1002/cam4.5802

**Published:** 2023-03-20

**Authors:** Sarah J. Storr, Victoria Hoskin, Radhika Aiyappa‐Maudsley, Abdi Ghaffari, Sonal Varma, Andrew Green, Emad Rakha, Ian O. Ellis, Peter A. Greer, Stewart G. Martin

**Affiliations:** ^1^ Nottingham Breast Cancer Research Centre, Biodiscovery Institute University of Nottingham, School of Medicine Nottingham UK; ^2^ Division of Cancer Biology and Genetics, Queen's Cancer Research Institute Queen's University Kingston Ontario Canada; ^3^ Department of Pathology and Molecular Medicine Queen's University Kingston Ontario Canada

**Keywords:** breast cancer, EZR, ezrin, VIL2

## Abstract

**Introduction:**

The cytoskeletal protein ezrin is upregulated in many cancer types and is strongly associated with poor patient outcome. While the clinical and prognostic value of ezrin has been previously evaluated in breast cancer, most studies to date have been conducted in smaller cohorts (less than 500 cases) or have focused on specific disease characteristics. The current study is the largest of its kind to evaluate ezrin both at the protein and mRNA levels in early‐stage breast cancer patients using the Nottingham (*n* = 1094) and METABRIC (*n* = 1980) cohorts, respectively.

**Results:**

High expression of ezrin was significantly associated with larger tumour size (*p* = 0.027), higher tumour grade (*p* < 0.001), worse Nottingham Prognostic Index prognostic group (*p* = 0.011) and HER2‐positive status (*p* = 0.001). High ezrin expression was significantly associated with adverse survival of breast cancer patients (*p* < 0.001) and remained associated with survival in multivariate Cox‐regression analysis (*p* = 0.018, hazard ratio (HR) = 1.343, 95% confidence interval (CI) = 1.051–1.716) when potentially confounding factors were included. High ezrin expression was significantly associated with adverse survival of patients whose tumours were categorised as receptor (oestrogen receptor (ER), progesterone receptor (PgR) or HER2) positive (*p* < 0.001) in comparison to those categorised as triple‐negative breast cancer (*p* = 0.889). High expression of ezrin mRNA (*VIL2)* in the METABRIC cohort was also significantly associated with adverse survival of breast cancer patients (*p* < 0.001).

**Conclusion:**

Retrospective analyses show that ezrin is an independent prognostic marker, with higher expression associated with shortened survival in receptor‐positive (ER, PgR or HER2) patients. Ezrin expression is associated with more aggressive disease and may have clinical utility as a biomarker of patient prognosis in early‐stage breast cancer.

## Graphical abstract

Ezrin expression is associated with clinical outcome of breast cancer patients.

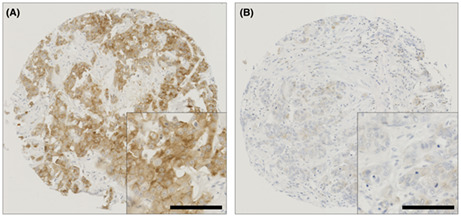

## 1 INTRODUCTION

Ezrin is a highly conserved protein, encoded by the gene known as *VIL2* or *EZR*, and functions as an important member of the ezrin/radixin/moesin (ERM) family of cytoskeleton‐associated proteins; mediating various dynamic cellular processes including cell morphology, motility and survival.^1^, ^2^ Ezrin is the most widely studied ERM protein and was originally discovered as a critical component of brush border epithelium of the kidney, placenta and intestine.^3^, ^4^ In non‐neoplastic tissue, expression and localisation of ezrin is tightly regulated and found predominantly at the apical surface; however, in the cancerous state, ezrin expression is significantly upregulated and is not only restricted to the apical membrane but also diffusely expressed in the cytoplasm.^5^ Overexpression of ezrin in cancer is presumably through transcriptional and/or epigenetic alterations, as no activating mutations for ezrin, to our knowledge, have been identified.

Many studies in different tumour types have investigated ezrin expression to determine if it has prognostic value, with meta‐analyses demonstrating that ezrin may be applicable as a prognostic marker in cancer patients with solid tumours; despite results from certain papers providing controversial results.^6^ The expression of ezrin in breast cancer patient tumours has been shown in a number of studies, including assessment between normal/benign breast lesions and cancer tissue, and/or associations with clinical outcome; these studies have often resulted in conflicting information. Expression of ezrin has been shown to be higher in malignant breast tissues^7^; other studies investigating ezrin expression in breast cancer tissue have focussed on its cellular distribution and how changes in distribution are associated with various tumour features.^8^ In terms of clinical outcome, a study of 443 breast cancer patients demonstrated that there was no association between native ezrin staining intensity and clinical outcome, but was significantly associated with recurrence when expression was normalised against matched normal tissue expression for each tumour core^9^; there have been further studies that have shown no significant association between patient survival and ezrin expression in unselected patient cohorts in 120 patients, and 347 patients.^5^, ^10^ A study of 487 breast cancer patient tumours demonstrated that high ezrin protein expression was associated with disease‐free survival,^11^ with another study of 117 breast cancer patients demonstrating that high ezrin expression was significantly associated with disease‐free survival and overall survival.^12^ In tumours from 377 breast cancer patients, high ezrin expression was significantly associated with adverse overall survival and disease‐free survival.^13^ Expression of ezrin mRNA has also been assessed in breast cancer, with high expression within the TCGA patient cohort associated with poor overall survival.^14^

Additional studies assessed ezrin expression in specific breast cancer subgroups. High ezrin expression has been significantly associated with disease‐free survival and overall survival in 134 node‐positive and high risk node‐negative breast cancer patients.^5^ High ezrin expression has also been shown to be significantly associated with shortened overall survival and progression‐free survival in a cohort of 249 triple receptor‐negative breast cancer patients.^15^ We sought, in the current study, to determine the frequency of ezrin mRNA and protein expression in large cohorts of well annotated unselected early‐stage invasive breast cancer patients and determine associations with patient survival.

## 2 METHODS

### 2.1 Nottingham cohort characteristics

Ethical approval for this study was obtained from Nottingham Research Ethics Committee 2, under the title ‘Development of a molecular genetic classification of breast cancer’ (C202313) and by North West – Greater Manchester Central Research Ethics Committee under the title ‘Nottingham Health Science Biobank (NHSB)’ (15/NW/0685). All samples collected from Nottingham used in this study were pseudo‐anonymised; those collected prior to 2006 did not require informed patient consent under the Human Tissue Act. All procedures performed in studies involving human participants were in accordance with the ethical standards of the institutional and/or national research committee and with the 1964 Helsinki declaration and its later amendments or comparable ethical standards.

In total. 1094 tumours from early‐stage invasive breast cancer patients were used, and patients were treated at Nottingham University Hospitals between 1987 and 1998. Early‐stage invasive breast cancer was defined as not having spread beyond the breast or the axillary lymph nodes and included Stage I, Stage IIA, Stage IIB and Stage IIIA breast cancers. All patients were managed in a standardised manner, where patients underwent wide local excision or mastectomy, which was decided by disease characteristics or patient choice. Following wide local excision or mastectomy, patients received radiotherapy if indicated. Patients received systemic adjuvant treatment on the basis of Nottingham Prognostic Index (NPI) values, oestrogen receptor (ER) and menopausal status. If patients had an NPI value less than 3.4 they did not receive adjuvant chemotherapy, whereas patients with an NPI value above 3.4 were candidates for CMF combination chemotherapy (cyclophosphamide, methotrexate and 5‐fluorouracil) if they were ER negative or premenopausal; and hormonal therapy if they were ER positive. The reverse Kaplan–Meier method was used to determine that the median follow‐up time was 201 months. Breast cancer‐specific survival was calculated as the time interval between primary surgery and death resultant from breast cancer. This study is reported according to reporting recommendations for tumour marker prognostic studies (REMARK) criteria. Clinicopathological information for this cohort is available in Table 1.

**TABLE 1:** Associations between ezrin protein expression, determined in 1094 early‐stage breast cancer patients using immunohistochemistry, with clinicopathological variables. The *p* values are resultant from Pearson's χ^2^ test of association and significant values (*p* < 0.05) are highlighted in bold.

	Ezrin expression		
	Low	High	*p* Value
Patient age			
<40 years	73 (6.7%)	24 (2.2%)	0.581
≥40 years	774 (70.8%)	222 (20.3%)	
Tumour size			
≤2 cm	519 (47.7%)	130 (11.9%)	**0.027**
>2 cm	326 (30.0%)	113 (10.4%)	
Tumour stage			
1	528 (48.5%)	141 (13.0%)	0.326
2	251 (23.1%)	77 (7.1%)	
3	66 (6.1%)	25 (2.3%)	
Tumour grade			
1	158 (14.5%)	27 (2.5%)	**<0.001**
2	298 (27.4%)	70 (6.4%)	
3	389 (35.8%)	146 (13.4%)	
Nottingham Prognostic Index (NPI)			
>3.4	275 (25.3%)	56 (5.2%)	**0.011**
3.4–5.4	425 (39.1%)	132 (12.2%)	
>5.4	144 (13.3%)	54 (5.0%)	
Vascular invasion			
Negative	476 (44.1%)	127 (11.8%)	0.226
Probable/definite	361 (33.5%)	115 (10.7%)	
Oestrogen receptor status			
Negative	217 (20.5%)	66 (6.2%)	0.738
Positive	601 (56.9%)	173 (16.4%)	
Progesterone receptor status			
Negative	345 (33.5%)	102 (9.9%)	0.760
Positive	446 (43.3%)	138 (13.4%)	
HER2 receptor status			
Negative	719 (67.4%)	192 (18.0%)	**0.001**
Positive	103 (9.7%)	52 (4.9%)	

### 2.2 METABRIC cohort characteristics

Information on the Molecular Taxonomy of Breast Cancer International Consortium (METABRIC) data set (*n* = 1980) have been previously published.^16^ METABRIC samples were collected by five centres in the United Kingdom and Canada between 1977 and 2005 and were acquired with appropriate consent from the respective institutional review boards. DNA and RNA were isolated from samples and hybridised to the Affymetrix SNP 6.0 and Illumina HT‐12 v3 platforms for genomic and transcriptional profiling as previously described.^16^ Adjuvant chemotherapy was given to almost all ER‐negative and lymph node‐positive patients, whereas ER‐positive and/or lymph node‐positive patients did not receive that treatment. Patients with HER2‐positive tumours did not receive trastuzumab. The reverse Kaplan–Meier method was used to determine that 141 months was the median follow‐up time.

### 2.3 Immunohistochemistry

Tissue microarrays (Nottingham cohort) were constructed using single 0.6 mm cores that were taken from a representative tumour area that was assessed using haematoxylin and eosin‐stained sections marked up by a specialist breast cancer histopathologist. These tissue microarrays have been used for multiple other studies.^17^, ^18^, ^19^ Immunohistochemical staining was performed using a Novolink Polymer Detection kit (Leica Biosystems), which was used according to the manufacturer's instructions. Briefly, tissue was deparaffinised and rehydrated in xylene, ethanol and then water. Antigen retrieval was performed in a microwave (10 min at 750 W followed by 10 min at 450 W), using 0.01 mol L^−1^ sodium citrate buffer (pH 6.0). Blocking was achieved using Novolink Peroxidase Block and Novolink Protein Block, with intervening Tris‐buffered saline (TBS) washes. Primary antibody was mouse anti‐Ezrin clone 3C12 (Millipore‐Sigma; used at a dilution of 1:1000) and was incubated on the tissue at room temperature for 1 h. Following primary antibody incubation, tissue was washed with TBS before incubation with Novolink Post Primary solution, TBS and then Novolink Polymer Solution. The chromogenic substrate was 3,3′‐diaminobenzidine and tissue was counterstained with haematoxylin. Finally, tissue was dehydrated and fixed using ethanol and xylene, prior to mounting using DPX. In each staining run, positive and negative controls were included. Controls were comprised of composite sections comprising Grade 1 and 2 early‐stage invasive breast tumours; negative controls had primary antibody omitted from each staining run.

Stained slides were scanned using a Nanozoomer Digital Pathology Scanner (Hamamatsu Photonics), and slide scans were assessed at 200× magnification. A semi‐quantitative immunohistochemical *H* score was used to determine cytoplasmic ezrin expression. The staining intensity was assessed as none (0), weak (1), medium (2) or strong (3) within tumour cells and a percentage area of each staining intensity was determined. To calculate a *H*‐score for each tumour, the intensity score of each percentage area was multiplied and combined together to create a variable with a potential range of 0–300. Greater than 30% of tumour cores were double assessed, and both assessors were blinded to clinical outcome and each other's scores. The single measure intraclass correlation coefficient was above 0.7 (0.734), which indicates good concordance between scorers.

### 2.4 Statistical analyses

Statistical analysis was performed using IBM SPSS Statistics (version 26). Cases were stratified based on breast cancer‐specific survival using X‐Tile software^20^ for protein expression, and around the median for mRNA expression. Spearman's rank test was performed to assess for correlations between protein or mRNA expression levels. The Pearson's χ^2^ test of association was used to determine the relationship between categorised protein and mRNA expression and clinicopathological variables. Survival curves were plotted according to the Kaplan–Meier method with significance determined using the log‐rank test. Multivariate survival analysis used the Cox proportional hazards regression model with multiple variables included simultaneously. All differences were deemed statistically significant at the level of *p* ≤ 0.05. Broad Institute Morpheus software was used to visualise data (https://software.broadinstitute.org/morpheus).

## 3 RESULTS

### 3.1 Ezrin protein staining: location and frequency

**FIGURE 1:**
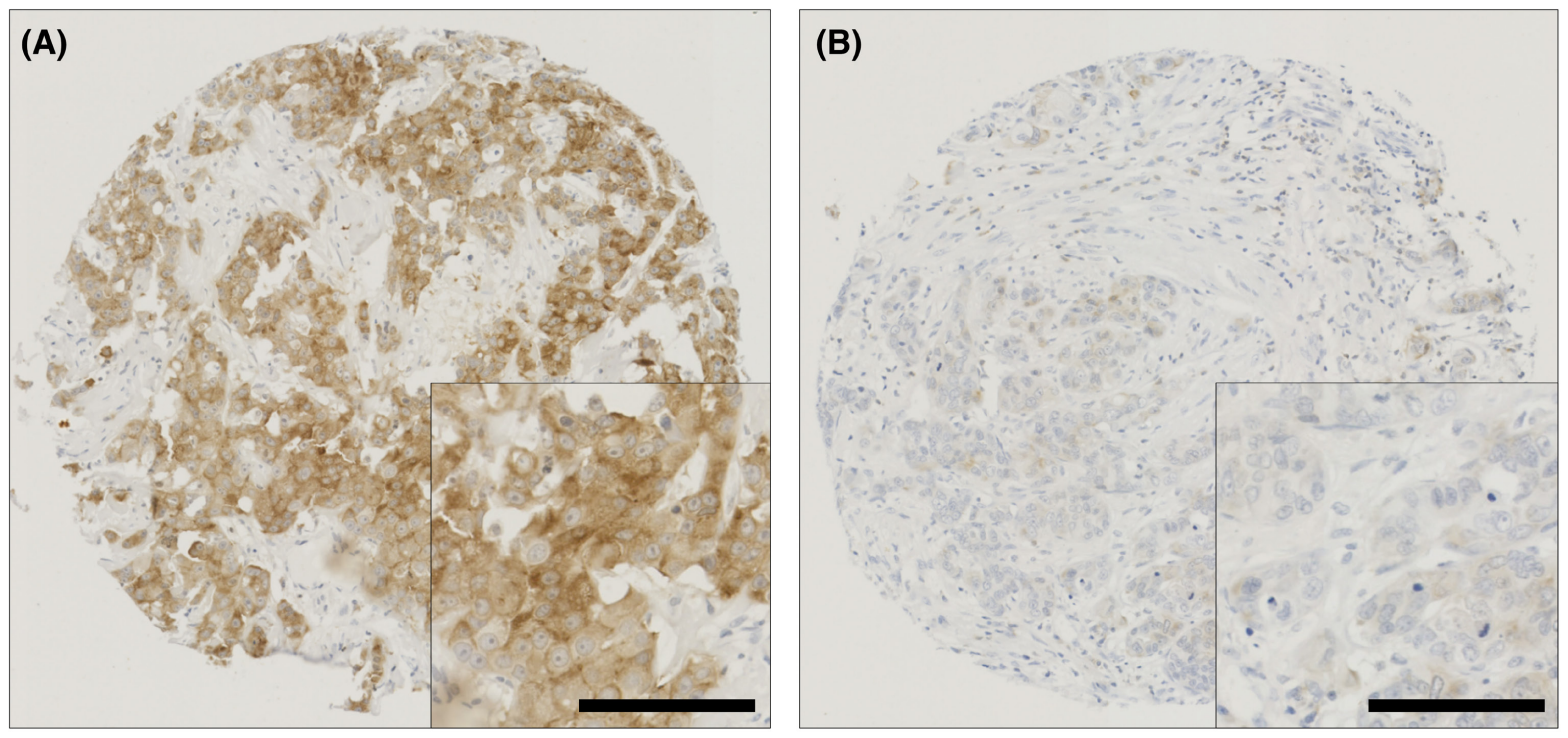
Representative photomicrographs of ezrin protein staining in high (A) and low (B) ezrin expressing tissues are shown at 10× magnification with a 20× magnification inset box. Scale bar represents 100 μm.

In total, 1094 early‐stage invasive breast cancer tumours were available for scoring. Ezrin expression was cytoplasmic with weak to strong staining intensity observed in tumour cells; representative staining patterns are shown in Figure 1. The median ezrin *H*‐score was 75 and ranged from 0 to 300. X‐tile computed a *H*‐score cut point of 200 with 22.5% (246/1094) cases demonstrating high expression.

### 3.2 Relationship between ezrin protein expression and clinicopathological variables

High expression of ezrin was associated with larger tumour size (χ^2^ = 4.921, d.f. = 1, *p* = 0.027), higher tumour grade (χ^2^ = 16.290, d.f. = 2, *p* < 0.001), worse NPI prognostic group (χ^2^ = 8.991, d.f. =2, *p* = 0.011) and HER2‐positive status (χ^2^ = 11.675, d.f. = 1, *p* = 0.001). No associations were observed between any other clinicopathological variables and ezrin expression (Table 1).

### 3.3 Association between ezrin protein expression and survival

**FIGURE 2:**
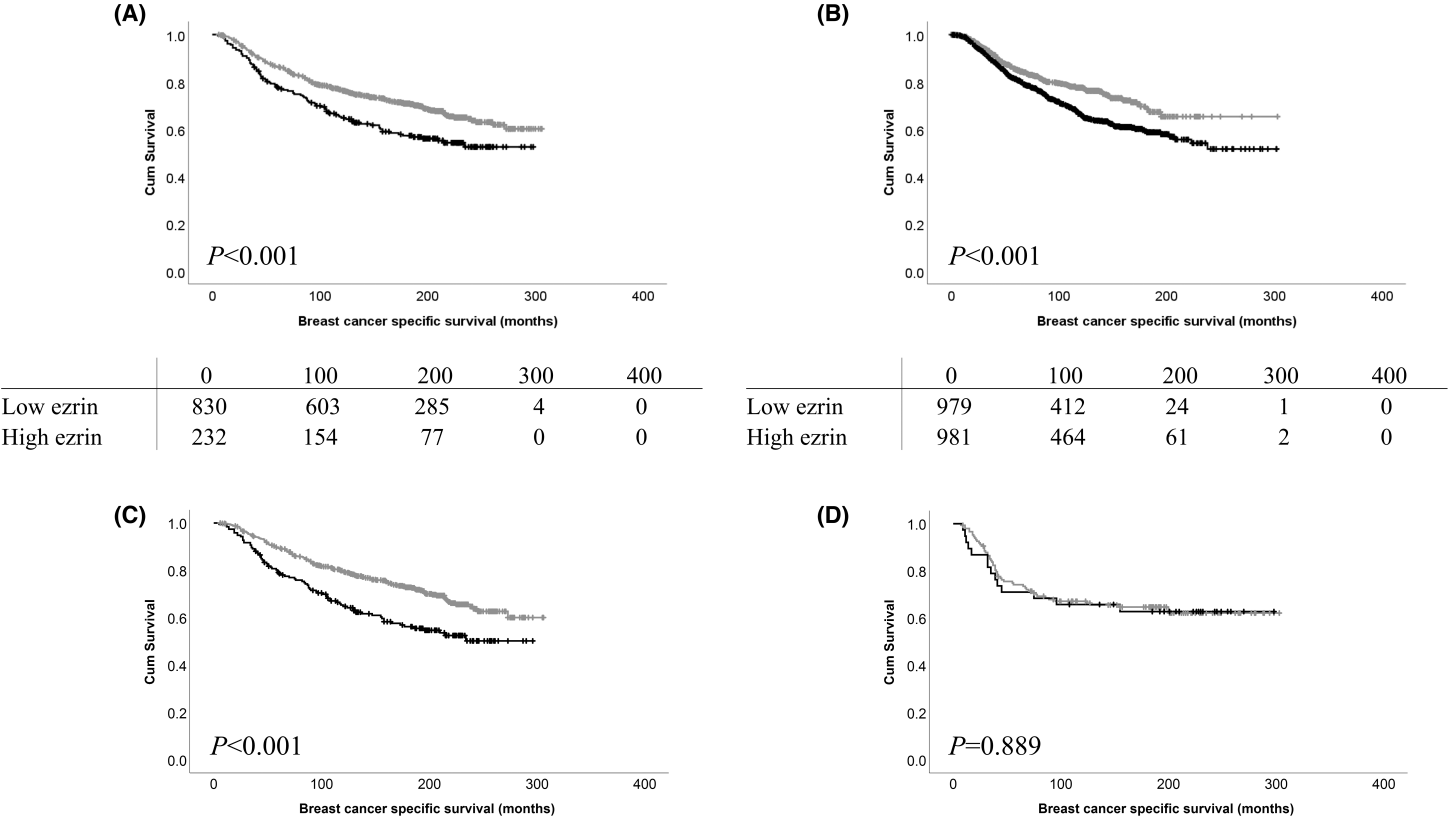
(A) Kaplan–Meier analysis of ezrin protein expression, and (B) *VIL2* mRNA expression where the relationship between breast cancer‐specific survival and low (grey line) or high (black line) expression are shown. The numbers shown below the Kaplan–Meier survival curves are the number of patients at risk at the specified month; these numbers demonstrate the number of cases available for assessment and therefore also indicate the number of cases with missing information. (C) Kaplan–Meier analysis of ezrin protein expression in non‐triple‐negative and (D) triple‐negative breast cancer where the relationship between breast cancer‐specific survival and low (grey line) or high (black line) expression are shown. Hazard ratios for the respective analyses are (A) = 1.513, (B) = 1.454, (C) = 1.699, and (D) = 1.043.

High ezrin expression was significantly associated with adverse survival of breast cancer patients (*p* < 0.001) (Figure 2A). Ten‐year survival of breast cancer patients with low expression of ezrin was 76.5% (95% confidence interval (CI) = 0.736–0.794), while survival for those with high expression of ezrin was 64.7% (95% CI = 0.584–0.710). High ezrin expression remained associated with survival in multivariate Cox‐regression analysis (*p* = 0.018, hazard ratio (HR) = 1.343, 95% CI = 1.051–1.716) when the potential confounding factors of tumour size, tumour stage, tumour grade, vascular invasion, ER status, progesterone receptor status (PgR) and HER2 status were included (all with individual Kaplan–Meier log‐rank statistics of *p* < 0.001) (Table 2A).

**TABLE 2:** Multivariate Cox regression analysis showing ezrin protein expression, various pathological variables and their effect upon disease‐specific survival in breast cancer patients (A), and in hormone receptor‐positive patients (B). (C) Multivariate Cox regression analysis showing *VIL2* expression, various pathological variables and their effect upon disease‐specific survival.

			95% CI	
	*p*‐Value	Hazard ratio	Lower	Upper
A				
Ezrin expression	0.018	1.343	1.051	1.716
Tumour size	0.022	1.311	1.041	1.653
Tumour stage	<0.001	1.923	1.628	2.272
Tumour grade	<0.001	1.549	1.270	1.890
Vascular invasion	0.001	1.501	1.178	1.913
ER status	0.109	1.312	0.941	1.830
PgR status	0.003	0.637	0.475	0.854
HER status	0.008	1.478	1.109	1.968
B				
Ezrin expression	0.007	1.435	1.103	1.868
Tumour size	0.133	1.216	0.942	1.570
Tumour stage	<0.001	1.964	1.635	2.360
Tumour grade	<0.001	1.842	1.514	2.241
Vascular invasion	0.006	1.456	1.117	1.899
C				
*VIL2* expression	<0.001	1.400	1.161	1.689
Tumour size	<0.001	1.904	1.571	2.308
Grade	0.003	1.289	1.088	1.527
PgR status	0.006	0.733	0.587	0.914
HER status	<0.001	1.518	1.196	1.927
ER status	0.023	0.755	0.592	0.962

### 3.4 Association between ezrin protein expression and survival in receptor‐positive breast cancer

High ezrin expression was significantly associated with adverse survival of patients whose tumours were categorised as receptor (ER, PgR or HER2) positive (*p* < 0.001; Figure 2C) in comparison to those categorised as triple‐negative breast cancer (*p* = 0.889; Figure 2D). High ezrin expression remained associated with adverse survival in the breast cancer subgroup using multivariate Cox‐regression analysis (*p* = 0.007, HR = 1.435, 95% CI = 1.103–1.868) when the potential confounding factors of tumour size, tumour stage, tumour grade and vascular invasion were included (all with individual Kaplan–Meier log‐rank statistics of *p* < 0.001) (Table 2B).

### 3.5 High VIL2 mRNA expression is associated with adverse patient survival

Ezrin mRNA (*VIL2*) expression was assessed against survival in the METABRIC dataset (*n* = 1980) (ILMN probe ILMN_1795937). Median *VIL2* expression was 11.64998142, ranging between 8.902730628 and 13.92283998; and expression was categorised around the median value. High expression of *VIL2* was significantly associated with adverse survival of breast cancer patients (*p* < 0.001) (Figure 2B). High *VIL2* expression remained associated with adverse survival in the breast cancer subgroup using multivariate Cox‐regression analysis (*p* < 0.001, HR = 1.400, 95% CI = 1.161–1.689) when the potential confounding factors of tumour size, tumour grade, ER status, PgR status and HER2 status were included (all with individual Kaplan–Meier log‐rank statistics of *p* < 0.001) (Table 2C).

High *VIL2* expression was associated with larger tumour size (χ^2^ = 8.474, d.f. = 1, *p* = 0.004), higher tumour grade (χ^2^ = 10.205, d.f. = 2, *p* = 0.006), HER2‐positive tumours (χ^2^ = 9.928, d.f. = 1, *p* = 0.002), ER‐positive tumours (χ^2^ = 34.598, d.f. = 1, *p* < 0.001) and PAM50 subtype (χ^2^ = 61.481, d.f. = 4, *p* < 0.001) (Table 3).

**TABLE 3:** Associations between *VIL2* mRNA expression with clinicopathological variables. The *p* values are resultant from Pearson's χ^2^ test of association and significant values (*p* < 0.05) are highlighted in bold.

	*VIL2* expression		
	Low	High	*p*‐Value
Tumour size			
≤2 cm	463 (23.6%)	396 (20.2%)	**0.004**
>2 cm	520 (26.5%)	580 (29.6%)	
PAM50 subtype			
Basal	214 (10.8%)	117 (5.9%)	**<0.001**
HER2	99 (5.0%)	239 (12.1%)	
Luminal A	342 (17.3%)	715 (36.2%)	
Luminal B	209 (10.6%)	490 (24.8%)	
Normal	126 (6.4%)	73 (3.7%)	
Tumour grade			
1	104 (5.5%)	65 (3.4%)	**0.006**
2	372 (19.7%)	398 (21.0%)	
3	467 (24.7%)	485 (25.6%)	
Nottingham Prognostic Index (NPI)			
>3.4	353 (17.8%)	328 (16.6%)	0.472
3.4–5.4	543 (27.4%)	1100 (55.6%)	
>5.4	95 (4.8%)	199 (10.1%)	
Oestrogen receptor status			
Negative	292 (14.7%)	180 (9.1%)	**<0.001**
Positive	699 (35.3%)	809 (40.9)	
Progesterone receptor status			
Negative	480 (24.2%)	458 (23.1%)	0.343
Positive	511 (25.8%)	531 (26.8%)	
HER2 receptor status			
Negative	891 (45.0%)	843 (42.6%)	**0.002**
Positive	100 (5.1%)	146 (7.4%)	

### 3.6 VIL2 mRNA expression and pathway associations

**FIGURE 3:**
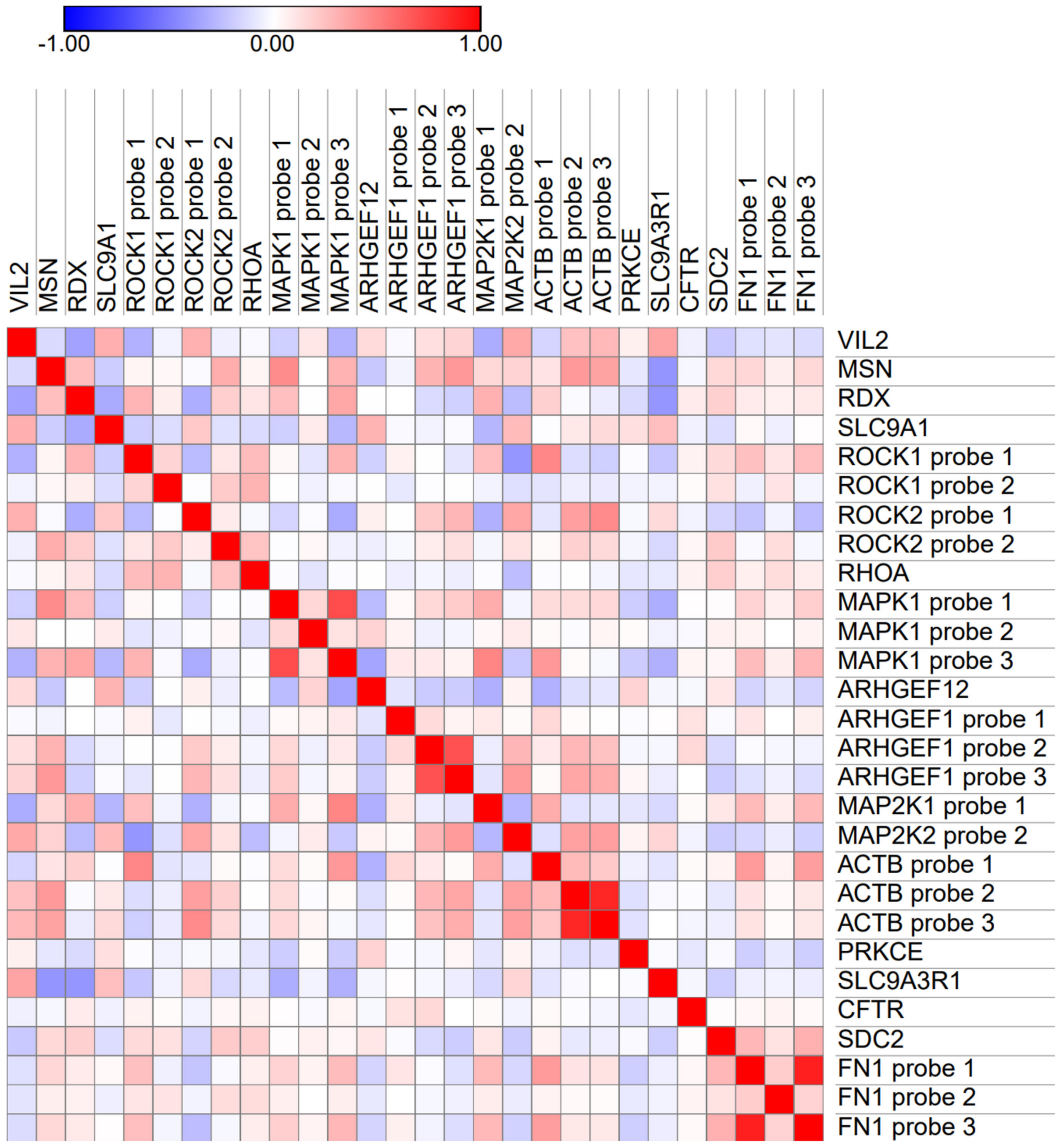
Similarity matrix of *VIL2*, *MSN*, *RDX*, *SLC9A1*, *ROCK1*, *ROCK2*, *RHOA*, *MAPK1*, *ARHGEF12*, *ARHGEF1*, *MAP2K1*, *MAP2K2*, *ACTB*, *PRKCE*, *SLC9A3R1*, *CFTR, SDC2* and *FN1* expression using Spearman's correlation coefficients (represented in the coloured scale bar).

In the Kyoto Encyclopaedia of Genes and Genomes (KEGG) database, *VIL2* is identified in pathways associated with the regulation of the actin skeleton (ko04810), tight junctions (ko04670) and proteoglycans in cancer (ko05205). Up‐ and down‐stream pathway partners were assessed in a similarity matrix (Figure 3) to understand the relationship between mRNA expression of pathway partners. The genes *VIL2, MSN* (ILMN_1659895)*, RDX* (ILMN_1708611)*, SLC9A1* (ILMN_1800425)*, ROCK1* (two probes available: ILMN_1808768 and ILMN_1739583), *ROCK2* (two probes available: ILMN_2058337 and ILMN_1659099), *RHOA* (ILMN_1781290)*, MAPK1* (three probes available: ILMN_2235283, ILMN_1706677 and ILMN_1767320), *ARHGEF12* (ILMN_1810712)*, ARHGEF1* (three probes available: ILMN_2293131, ILMN_2405129, and ILMN_1772370), *MAP2K1* (two probes available: ILMN_1694240 and ILMN_1657968), *ACTB* (three probes available: ILMN_1777296, ILMN_2038777 and ILMN_2152131), *PRKCE* (ILMN_1717799)*, SLC9A3R1* (ILMN_1680925)*, CFTR* (ILMN_1705813)*, SDC2* (ILMN_1784553)*, FN1* (three probes available: ILMN_2366463, ILMN_1675646 and ILMN_1778237) were assessed. Genes with expression linked to *VIL2* with a *R* ^2^ value greater than 0.3 or − 0.3, were *RDX* (*R* ^2^ = −0.365, *p* < 0.001), *SLC9A1* (*R* ^2^ = 0.313, *p* < 0.001), *ROCK1* probe 1 (*R* ^2^ = −0.305, *p* < 0.001), *ROCK2* probe 1 (*R* ^2^ = −0.311, *p* < 0.001), *MAPK1* probe 3 (*R* ^2^ = −0.302, *p* < 0.001), *MAP2K2* probe 1 and probe 2 (*R* ^2^ = −0.325, *p* < 0.001 and *R* ^2^ = −0.336, *p* < 0.001, respectively).

## 4 DISCUSSION

High ezrin protein expression was significantly associated with shortened survival in a large cohort of early‐stage breast cancer patients. Importantly, this remained so in multivariate analysis when potentially confounding prognostic variables were included. Interestingly, high ezrin expression was significantly associated with adverse survival of patients that had receptor‐positive disease, in comparison to no association with survival in patients with receptor‐negative disease. Previous studies have investigated ezrin protein expression in smaller cohorts of breast cancer patients, or patients with specific disease characteristics, such as triple‐negative disease, to show associations with clinical outcomes.^5^, ^14^, ^15^ Although high ezrin expression has been associated with featured of poor prognosis, links with disease‐specific survival have not always been demonstrated; this has been the case in studies with patient cohorts of *n* = 443,^9^ *n* = 120 patients and *n* = 347 patients.^5^, ^10^ Ezrin has been associated with some measures of clinical outcome in other studies, including association with disease‐free survival in *n* = 487 patients,^11^ and both disease‐free and overall survival in *n* = 117 patients.^12^ This is the largest study to determine ezrin protein expression in breast cancer to date. In addition to assessing protein expression, this study also demonstrated that high ezrin mRNA expression (*VIL2*) was associated with shortened disease‐specific survival of breast cancer patients using the METABRIC cohort, which supports our results at the protein level. Expression of ezrin mRNA has been previously assessed in breast cancer, with high expression within the TCGA patient cohort associated with poor overall survival,^14^ in support of these findings. Ezrin may prove to be an interesting biomarker in breast cancer and future work should investigate larger, more geographically diverse patient cohorts, including standardisation of cut points.

This study demonstrated that high ezrin expression was significantly associated with adverse survival of patients that had receptor‐positive disease, in comparison to no association with survival in patients with receptor‐negative disease. Direct links between ezrin and hormone receptor signalling in breast cancer have been reported in vitro previously; in particular, links between ezrin activation following 17β‐oestradiol (E2) treatment have been observed.^21^, ^22^ Ezrin has also been shown to co‐localise and interact with HER2 to maintain active HER2 at the cell surface in vitro.^23^ That this study was not able to assess ezrin activity in addition to ezrin protein and mRNA expression, is a limitation; however, this study supports previous findings that indicate a role for ezrin in receptor‐positive disease. Future work will continue to expand upon these findings in vitro.

In addition to patient survival, ezrin protein and mRNA expression were also associated with a number of important clinicopathological criteria. Ezrin mRNA and protein expression were both associated with larger tumour size, higher tumour grade and HER2‐positive tumours. Ezrin protein expression was associated with worse NPI prognostic group, whereas ezrin mRNA expression was associated with ER‐positive tumours and PAM50 subtype. Associations between HER2 status, tumour grade and ER status and ezrin expression, have been described previously; however, this is within the context of specific localisation patterns of ezrin.^8^ An association between high ezrin expression and ER‐ and PgR‐negative tumours and tumours that were larger, or at a more advanced stage, has also been demonstrated,^11^ in addition to an association with lymph node metastasis; a further study has also demonstrated a similar association.^12^ The finding that high ezrin expression is associated with clinicopathological criteria linked with adverse prognosis was somewhat expected, given the link between ezrin and patient survival.

Links between ezrin mRNA expression and genes identified in related pathways were explored as part of this study to determine how expression was linked in patient tumours. Genes linked to *VIL2* expression in breast cancer with a *R* ^2^ value greater than 0.3 or − 0.3, were *RDX*, *SLC9A1*, *ROCK1* probe 1, *ROCK2* probe, *MAPK1* probe 3, and *MAP2K2* probe 1 and probe 2. In some cases, differences between the expression of all probes were noted; this would require further downstream assessment to understand links between probes and transcript expression. Links between expression of these signalling partners were not unexpected; however, these findings may warrant further investigation and verification.

In conclusion, this study demonstrates that high ezrin protein expression is associated with shorter patient survival in a large, well‐annotated early‐stage invasive breast cancer patient cohort. Interestingly, a stronger association between ezrin expression and patient survival was observed in receptor‐positive disease. This finding was also observed at the mRNA level, with high *VIL2* expression significantly associated with shorter survival of patients in the METABRIC cohort. This data supports the role of ezrin as a prognostic marker with potential clinical utility in breast cancer.

## AUTHOR CONTRIBUTIONS

**Sarah J Storr:** Conceptualization (supporting); data curation (equal); formal analysis (equal); methodology (equal); project administration (equal); supervision (equal); writing – original draft (equal). **Victoria Hoskin:** Conceptualization (supporting); data curation (equal); formal analysis (equal); investigation (equal); writing – review and editing (equal). **Radhika Aiyappa‐Maudsley:** Investigation (equal); writing – review and editing (equal). **Abdi Ghaffari:** Formal analysis (equal); writing – review and editing (equal). **Sonal Varma:** Formal analysis (equal); writing – review and editing (equal). **Andrew Green:** Resources (equal); writing – review and editing (equal). **Emad Rakha:** Resources (equal); writing – review and editing (equal). **Ian Ellis:** Resources (equal); writing – review and editing (equal). **Peter A. Greer:** Conceptualization (equal); project administration (equal); supervision (equal); writing – original draft (equal). **Stewart Martin:** Conceptualization (equal); funding acquisition (equal); project administration (equal); resources (equal); writing – original draft (equal).

## FUNDING INFORMATION

S.J.S. is funded through the University of Nottingham's Research Vision as a Nottingham Research Fellow. V.H. was funded by MITACS fellowships (#17055 and #18854) in partnership with Tika Therapeutics Inc. The ezrin research program at Queen's University is supported by grants from the Canadian Cancer Society (#707329 and #705768), Canadian Institutes of Health Research (CCP‐160398) and the Ontario Institute of Cancer Research (P.CTIP.834).

## CONFLICT OF INTEREST STATEMENT

No conflict of interests are declared.

## Data Availability

Data sharing of the METABRIC data is not applicable to this article as it was not created in this study. Immunohistochemistry data will be provided upon request.
